# Inferior thyroid artery pseudoaneurysm associated with internal jugular vein puncture: a case report

**DOI:** 10.1186/s12871-015-0052-6

**Published:** 2015-05-06

**Authors:** Jinguang Ruan, Cao Zhang, Zhiyou Peng, David Yue Tang, Zhiying Feng

**Affiliations:** 1 Department of Anesthesiology and Pain Medicine, the First Affiliated Hospital, Zhejiang University School of Medicine, Hangzhou, China; 2 Department of Anesthesiology, the Fourth Affiliated Hospital, Zhejiang University School of Medicine, Yiwu, China; 3 Department of Anesthesiology, Mercy General Hospital, Sacramento, CA USA

**Keywords:** Pseudoaneurysm, Internal jugular vein, Inferior thyroid artery

## Abstract

**Background:**

Central venous catheter placement is an important aspect of patient care for the administration of fluids and medications and for monitoring purposes. However, it is still associated with significant morbidity and mortality.

**Case presentation:**

We report a case of iatrogenic inferior thyroid artery pseudoaneurysm during the central line placement due to internal jugular vein puncture. This is a rare complication of central venous cannulation. Fortunately the pseudoaneurysm was monitored closely, diagnosed promptly and obliterated by using radiological intervention. We discuss the risk factors and management of the unintended artery puncture.

**Conclusion:**

The pathway of the management post arterial puncture depends on the size of the needle or catheter, which is direct related to the consequence of arterial injuries. Identifying risk factors is very important to avoid the complications. However, the use of ultrasound guided venipuncture is the most important method to avoid mechanical complications.

## Background

The safety and advantages of Seldinger technique for central vein catheter (CVC) placement are well known. However, it is still associated with significant morbidity and mortality. Arterial injury is not uncommon during CVC placement and potentially fatal. Most common arterial puncture is involved in carotid artery [1], but also in vertebral artery [2], thyrocervical trunk [3], innominate artery [4], even internal mammary artery [5] etc. Some literatures showed inadvertent arterial puncture with a small needle occurs in range of 4.2-9.3% [6-9]. The most devastating complications from arterial misplacement of large-bore catheter (>7 Fr) have an incidence of 0.1 to 1.0% [1,9-12]. The hematoma in the neck, which can potentially compress the airway [5], hemothorax [13,14], pseudoaneurysm [14], arteriovenous fistula [15], stroke [10,16-19] and death [11,20,21] have been well reported. Here we report a case the patient scheduled for elective partial hepatectomy developed inferior thyroid artery pseudoaneurysm during CVC placement using Seldinger’s technique.

## Case presentation

The Case Report was approved by the ethics committee of the First Affiliated Hospital, Zhejiang University School of Medicine and the patient in this study was given written informed consent for her participation. A 51-year old Chinese woman (158 cm, 61 kg) presented with history of primary hepatocellular carcinoma with liver cirrhosis. Elective right-sided hepatectomy was scheduled. She denied any past surgical histories and had no anomalies on the neck in physical examination. All the vital signs were normal. Platelet counts and liver function were within normal range. The International Normalized Ratio (INR) was 1.35 (normal range: 0.85-1.15). The electrocardiogram and chest X-ray showed insignificantly findings prior to surgery.

After radial artery catheterization was placed, general anesthesia was induced with midazolam 2 mg, etomidate 16 mg, lidocaine 60 mg, and fentanyl 0.3 mg. Patient was intubated with rocuronium 50 mg on controlled ventilation. A 500 ml saline bag was placed under the right shoulder. Patient was placed in Trendelenburg position and her head was tilted to the left about 40 degree. The right neck was prepared and draped for CVC insertion with high central approach. After the finder needle penetrated obliquely into skin towards the right nipple until blood return, an 18-gauge introducer needle with 10 ml spring-wire introduction syringe (Arrow® central venous catheter, Arrow International, Asheboro, NC, USA) was inserted to the same site with the similar angle and similar direction as the finder needle did. However, the bright, pulsatile blood was observed during aspiration. The introducer needle was pulled out immediately and local compression was applied for hemostasis. After about 15 min manual compression, a diffused swelling was noted at the base of the right neck. Then CVC via the left internal jugular vein puncture was achieved successfully under the real-time guidance of the ultrasound at one attempt (Arrow® central venous catheter, 7Fr, 20 cm, 0.32 inches, two lumens; Arrow International, Asheboro, NC, USA). The surgery proceeded smoothly and the patient’s swelling in right neck was noted without any pulsation and bruit while transporting to ward.

**Figure 1:**
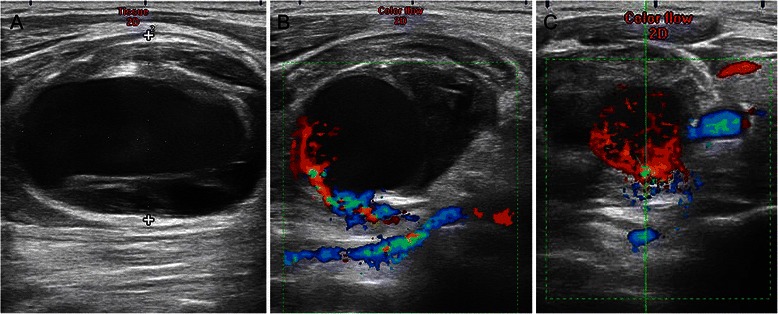
Radiological imaging. **A**: The cervical cystic mass (5*3.2 cm) was located at the right neck; **B**: The cervical cystic mass was linked with a near artery; the size of orificium fistulae was about 0.2 cm, and **C** showed the flow velocity reached at 0.68 m/s.

**Figure 2:**
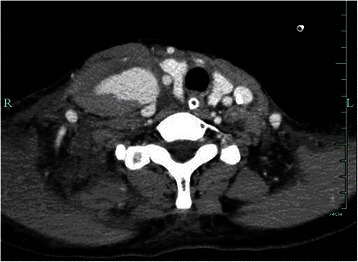
Computed Tomography Carotid Angiography showing the higher density mass (3.6*5.9 cm) of right neck connected with a branch of the subclavicular artery and compressed the right carotid artery and trachea.

**Figure 3:**
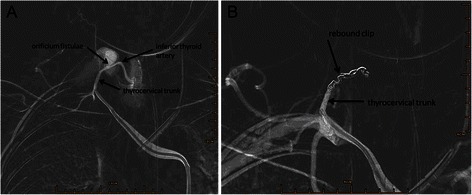
Radiological imaging. **A** showing a pseudoaneurysm fed from the inferior thyroid artery; **B** showing the embolization the orificium fistulae of the right inferior thyroid with a cook clip, no more hemorrhage was observed under digital subtraction angiography.

**Figure 4:**
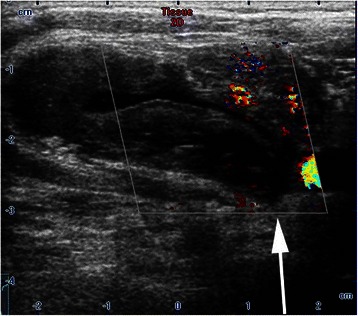
The ultrasound re-scanning showed the right cervical cystic mass without pulsative hemorrhage after embolization.

On postoperative Day (POD) 1, the swelling was still noticeable at the right neck without any signs of pulsation and bruit. Physical compression about 1 h was applied again. However, the maneuver did not reduce swelling on POD2, and the patient complained of a mild respiratory distress. The emergent Doppler ultrasound and Computed Tomography Angiography were recommended. The ultrasound results (Figure 1) showed that the cervical cystic mass (5*3.2 cm) was located at the right neck and linked with a near artery; the size of orificium fistula was about 0.2 cm, and the flow velocity reached at 0.68 m/s. The computed tomography scan of the neck demonstrated a giant hematoma compressing the internal jugular vein and trachea (Figure 2). After consulting with vascular surgeon, right subclavian artery angiography guided by digital subtraction angiography was immediately ordered about 58 hours postoperatively. A pseudoaneurysm arising from the inferior thyroid artery (Figure 3A) was verified and its orificium fistula was embolized by cook clipHilal/Nester® Embolization Microcoil, MWCE- 18–2.0-2- HILAL /MWCE-18- 14-3-NESTER, Cook Incorporated, Bloomington, USA) (Figure 3B). The procedure was successful by the confirmation of the ultrasound re-scanning without any abnormal flow (Figure 4). The patient remained stable, and was discharged on POD 10 without any sequelae.

## Discussion

After reviewing the various complications of arterial puncture during CVC placement, we found that the majority of the devastating complications in arterial injuries were reported on carotid or vertebral with large-bore catheters [21], rare on small caliber artery like inferior thyroid artery. Only one case report on inferior thyroid artery injury with focal false aneurysm after multiple attempts of CVC placement with 22 gauge finder needle. The patient had platelet abnormality. The false aneurysm of inferior thyroid artery was embolized with coils successfully [22]. Another case reported on unintended inferior thyroid artery cannulation during CVC placement with 8.5 FG introducer sheath. In this case, 71 year old male patient with liver carcinoma for left-sided hemi-hepatectomy, vascular surgeon immediately explored the neck and found an enlarged and punctured inferior thyroid artery, which was ligated without further complications [23].

However, in our case, the arterial puncture with 18 gauge introducer needle was made by a 3rd year resident who was supervised by a senior attending with only one attempt. We postulate that the formation of inferior thyroid artery pseudoaneurysm could be due to following factors. First, the introducer needle was aimed too lateral to carotid artery, even lateral to internal jugular vein, or too low and deep in the neck. Second, the direct compression was applied to the needle entry site on the skin, but maybe not on the punctured site on the artery. Third, an arterial puncture usually results in a periarterial hematoma and is usually self-limiting with application of pressure. However, if the injury to the artery is severe or if coagulation abnormalities exist, a false aneurysm can be produced. Coagulopathy contributed to the formation of hematoma, arteriorial venous fistula, and pseudoaneurysm had been well reported [24-27]. Even blood liver function tests were normal in this patient, the INR was prolonged slightly prior to surgery. And the prothrombin time does not become abnormal until more than 80% of liver synthetic capacity is lost [28]. INR was not measrued unfornately after surgery during POD1 and POD3. The patient’s liver synthetic function might further be compromised by showing abnormal alanine aminotransferase (ALT) and aspartate aminotransferase (AST), bilirubin and albumin on POD1. Those abnormal values resumed to normal on postoperative day 3. Therefore, the patient’s coagulopathy aggravated during and post surgery. Insufficient clotting factors may theoretically have an increased risk for a delayed closure of an arterial puncture. All those factors above might be the explanations for the formation of pseudoaneurysm of inferior thyroid artery for this case.

Clinical investigation revealed that both small needle and large-bore catheter could result in arterial injuries during CVC cannulation attempts. The management or consequence of arterial punctures is directly related to the size of needle or catheter during CVC placement. The definition of the large-bore catheter is mentioned in some literatures, which indicate unintended arterial cannulation with a 7 French or larger catheter or dilator [6,21,29]. There are no definite guidelines about the management of accidental arterial cannulation during central venous catheterization. We concur with Marie-Christine Guilbert et al., their recommendation for the management [21]. As soon as operator suspects the large-bore catheter (>7 F) in artery, leaves the catheter in place, obtain vascular surgery consultation, and postpones any elective surgery. The definition of small needle is from 20-G to 25-G by reviewing literatures [21]. Accidental arterial punctures caused by those finder needles are usually inconsequential. The pull/pressure technique is acceptable if there are no risk factors [11]. However, small finder needle with multiple attempts on the condition of the arterosclerosis or coagulopathy still can resulted in major complications, such as stroke, pseudoaneurysm, etc. [10,22,30,31]. In present case, we fell in the dilemma. No literatures or the consensus define 18 gauge needle as small caliber needle or large-bore cannula. No guideline of management of 18-G needle on arterial puncture is found. The pull/pressure technique was taken in our case. The patient developed acute inferior thyroid artery pseudoaneurysm within 3 days. The pull/pressure technique was used because we thought 18 G needle was not much traumatic, but the fact it was traumatic on the patients with high risk factors. The cirrhosis with coagulopathy as risk factor was neglected. No real-time ultrasonography was used during the CVC attempt; No Doppler ultrasound exam was done immediately post puncture while noticing neck swelling. The investigation should start early postoperatively. Those are the lessons we learn from this case.

By reviewing the literatures, anatomical variations, obesity, history of neck surgery and vessel cannulation, inexperience operator, extreme rotation of the neck, multiple needle attempts, large-bore needle, insertion too deep (>2 cm) or too laterally, coagulopathy, diseased artery, and low approach of Seldinger’s technique, etc., are described as risk factors for potential complications of iatrogenic arterial puncture [2,21,24,25,30]. Prevention of arterial injury has been focused on the trainee training and the application of ultrasonography during CVC placement. Meta-analysis of randomized controlled trials [32-42] indicates that, compared with the landmark-guided technique, real-time ultrasound guided venipuncture of the internal jugular vein has a higher first insertion attempt success rate, reduced access time, higher overall successful cannulation rate, and decreased rates of arterial puncture (Category A1 evidence). Though there are still several reports of inadvertent arterial placement of large-bore catheters that have occurred on some unusual locations despite the use of ultrasound guidance with some trainee [43,44].

## Conclusions

In summary, iatrogenic inferior thyroid artery pseudoaneurysm is infrequent complication during CVC placement. To our knowledge, there was only one case of the inferior thyroid artery focal pseudoaneurysm reported [22]. The pathway of the management post arterial puncture depends on the size of the needle or catheter, which is direct related to the consequence of arterial injuries. Identifying risk factors is very important to avoid the complications. However, the use of ultrasound-guided venipuncture is the most important method to avoid mechanical complications [45-48]. This case teaches us that ultrasound guided venipuncture MUST be used routinely. Though ultrasound machine is not well equipped in developing country, it is still highly recommended for real-time ultrasound guidance during the whole period of CVC placement, especially for high risk patients [45-48].

## Consent

Written informed consent was obtained from the patient for publication of this case report and any accompanying images. A copy of the written consent is available for review by the editor of this journal.

## Abbreviations

CVC: Central venous catheter
POD: Postoperative Day
INR: International Normalized Ratio
ALT: Alanine aminotransferase
AST: Aspartate aminotransferase
